# Contributions of mutations in *acrR* and *marR* genes to organic solvent tolerance in *Escherichia coli*

**DOI:** 10.1186/2191-0855-2-58

**Published:** 2012-11-13

**Authors:** Rei Watanabe, Noriyuki Doukyu

**Affiliations:** 1 Graduate School of Interdisciplinary New Science, Toyo University, 2100, Kujirai, Kawagoe, Saitama, 350-8585, Japan; 2 Bio-Nano Electronic Research Center, Toyo University, 2100, Kujirai, Kawagoe, Saitama, 350-8585, Japan; 3 Department of Life Science, Toyo University, 1-1-1 Izumino, Itakura-machi, Gunma, 374-0193, Japan

**Keywords:** Escherichia coli, Organic solvent tolerance, MarR, AcrR, AcrAB-TolC, Efflux pump

## Abstract

The AcrAB-TolC efflux pump is involved in maintaining intrinsic organic solvent tolerance in *Escherichia coli.* Mutations in regulatory genes such as *marR*, *soxR*, and *acrR* are known to increase the expression level of the AcrAB-TolC pump. To identify these mutations in organic solvent tolerant *E*. *coli*, eight cyclohexane-tolerant *E*. *coli* JA300 mutants were isolated and examined by DNA sequencing for mutations in *marR*, *soxR*, and *acrR*. Every mutant carried a mutation in either *marR* or *acrR*. Among all mutants, strain CH7 carrying a nonsense mutation in *marR* (named *marR109*) and an insertion of IS*5* in *acrR*, exhibited the highest organic solvent-tolerance levels. To clarify the involvement of these mutations in improving organic solvent tolerance, they were introduced into the *E. coli* JA300 chromosome by site-directed mutagenesis using λ red-mediated homologous recombination. Consequently, JA300 mutants carrying *acrR*::IS*5*, *marR109*, or both were constructed and named JA300 *acrR*IS, JA300 *marR*, or JA300 *acrR*IS *marR*, respectively. The organic solvent tolerance levels of these mutants were increased in the following order: JA300 < JA300 *acrR*IS < JA300 *marR* < JA300 *acrR*IS *marR*. JA300 *acrR*IS *marR* formed colonies on an agar plate overlaid with cyclohexane and *p*-xylene (6:4 vol/vol mixture). The organic solvent-tolerance level and AcrAB-TolC efflux pump-expression level in JA300 *acrR*IS *marR* were similar to those in CH7. Thus, it was shown that the synergistic effects of mutations in only two regulatory genes, *acrR* and *marR*, can significantly increase organic solvent tolerance in *E. coli*.

## Introduction

Whole-cell biocatalysts are beneficial in the biotransformation involved in their internal cofactor regeneration and in bioconversions requiring multi-step metabolic pathways. Whole-cell biocatalysts are available in two-phase systems consisting of organic solvent and an aqueous medium, that are potentially advantageous for the bioconversion of hydrophobic and/or toxic organic compounds (Schmid et al. 1998 ; Sardessai and Bhosle 2004 ; Heipieper et al. 2007 ). The use of a second organic phase improves productivity levels and product recovery, unlike the case with conventional media whose substrate solubility is poor. One of the main limitations in the application of whole-cell biocatalysts in two-phase systems is the instability of biocatalysts due to the toxicity of organic solvents toward the cells. When microorganisms are incubated in the presence of a large amount of an organic solvent, the extent of growth inhibition is inversely correlated with the log *P*_OW_ of the solvent ( Inoue and Horikoshi 1989 ). Hydrophobic organic solvents with a log *P*_OW_ of 2 to 5 bind to the cells and disrupt the cell membrane (Sikkema et al. 1994 ; Aono et al. 1994 ).

Various mechanisms underlying microbial tolerance and responses to solvents have been revealed by the genetic, physiological and biochemical characterization of organic solvent tolerant bacteria such as *Pseudomonas* species and *E*. *coli* mutants ( Aono 1998 ; Ramos et al. 2002 ; Shimizu et al. 2005 ; Heipieper et al. 2007 ; Okochi et al. 2007 ; Okochi et al. 2008a ; Okochi et al. 2008b ; Torres et al. 2011 ; Segura et al. 2012 ). Until now, more is known about how cells respond to organic solvents, but less about how to develop tolerant strains.

It has been shown that energy-dependent efflux pumps belonging to the resistance/nodulation/cell division (RND) family (Paulsen et al. 1996 ) are important for the maintenance of solvent tolerance in Gram-negative bacteria (White et al. 1997 ; Kieboom et al. 1998 ; Ramos et al. 1998 ; Li et al. 1998 ; Tsukagoshi and Aono 2000 ). In *E. coli*, the AcrAB-TolC efflux pump belonging to the RND family has been shown to provide intrinsic tolerance to organic solvents ( Tsukagoshi and Aono 2000 ). This pump enhances the release of solvents intracellularly accumulated in *E. coli* cells. *acrAB* and *tolC* are *marA/soxS/rob* regulon genes ( Barbosa and Levy 2000 ). MarA and SoxS proteins are transcriptional activators belonging to the AraC/XylS family ( Alekshun and Levy 1997 ). These activators control the expression of *marA/soxS/rob* regulon genes. MarA and SoxS are transcriptionally regulated. Transcription of the *marRAB* is repressed by MarR, whereas it is autoactivated by MarA. *soxS* transcription is repressed by SoxR and enhanced by the activated form of SoxR after exposure to superoxides or nitric oxide ( Pomposiello and Demple 2001 ). Mutations in *marR* or *soxR* were suggested to enhance the expression level of the AcrAB-TolC efflux pump (Okusu et al. 1996 ; Aono 1998 ; Randall and Woodward 2002 ; Komp Lindgren et al. 2003 ). In addition, *acrAB* expression is modulated locally by the repressor AcrR (Ma et al. 1996 ). Thus, mutations in *acrR* can lead to the enhanced expression of AcrAB. Mutations conferring a multidrug resistance phenotype have been found in the genes *marR*, *soxR*, and *acrR* among clinical and veterinary *E. coli* isolates (Wang et al. 2001 ; Webber and Piddock 2001 ; Komp Lindgren et al. 2003 ; Fernandes et al. 2003 ; Karczmarczyk et al. 2011 ). Some of those studies suggested that organic solvent tolerance is correlated with these mutations in these isolates (Wang et al. 2001 ; Komp Lindgren et al. 2003 ). However, the extent to which these mutations contribute to organic solvent tolerance has not been clarified because *E. coli* isolates used in these studies had a variety of genetic backgrounds. In addition, the synergistic effects of these mutations on organic solvent tolerance were ambiguous. To clarify the effects of mutations on the tolerance phenotype, it is necessary to reconstruct selected mutations in one type of strains in various combinations.

In this study, we isolated organic solvent-tolerant *E. coli* mutants and identified mutations in regulatory genes (*marR*, *soxR*, *acrR*). Among these mutants, we selected the one that exhibited the highest organic solvent tolerance and investigated the contributions of each identified mutation to organic solvent tolerance. As a result, we clarified that the *E. coli* strain can acquire high-level organic solvent tolerance due to mutations in only two regulatory genes (*marR* and *acrR*) by reconstructing these mutations in a parent strain. This study provides a new finding for developing an *E*. *coli* with high organic solvent tolerance.

## Materials and methods

### Materials, media, and culture conditions

The organic solvents used were of the highest quality available (Wako Pure Chemical Industries, Osaka, Japan). The purity of these solvents is more than 98%. The organisms were grown aerobically at 30°C in LBG medium consisting of 1% Bacto Tryptone (Difco Laboratories, Detroit, MI), 0.5% Bacto Yeast Extract (Difco), 1% NaCl, and 0.1% glucose. This medium supplemented with 10 mM MgSO_4_ (LBGMg medium) (Aono et al. 1991 ) was also used. The LBGMg medium was solidified with 1.5% (wt/vol) agar. Ampicillin (50 μg/ml) or kanamycin (50 μg/ml) was added to the medium when necessary. The organisms were also grown in LB medium, which is identical to LBG medium except that glucose is omitted.

### Bacterial strains and plasmids

*E. coli* K-12 derivatives used to evaluate solvent tolerance are summarized in Table 1. Strain JW0452, a BW25113-based *acrA*::Km^R^ (kanamycin resistant), was supplied by the National Bio-Resource Project (NIG, Mishima, Japan): *E. coli* (Baba et al. 2006 ).

**Table 1:** Bacterial strains and plasmids used

***E. coli *strain**	**Genotype**	**Reference**
JA300	F^-^*thr leuB6 trpC1117 thi rpsL20 hsdS*	Aono et al. 1991
JA300 *acrR*IS	Same as JA300, but with *acrR*::IS*5*	This study
JA300 *marR*	Same as JA300, but with *marR109*	This study
JA300 *acrR*IS *marR*	Same as JA300, but with *acrR*::IS*5* and *marR109*	This study
JA300Δ*acrA*	Same as JA300, but with *acrA*::Km^R^	This study
JA300Δ*acrA acrR*IS *marR*	Same as JA300, but with *acrA*::Km^R^, *acrR*::IS*5* and *marR109*	This study
BW25113	*lacI*^q^*rrnB*_T14_ lacZ_WJ16_*hsdR*514 *araBAD*_AH33_*rhaBAD*_LD78_	Baba et al. 2006
JW0452	Same as BW25113, but with *acrA*::Km^R^	Baba et al. 2006

### Isolation of cyclohexane-tolerant mutants of *E. coli* JA300

First, 100 μl of the overnight culture of strain JA300 grown in LBGMg medium was inoculated into 10 ml of fresh LBGMg medium overlaid with 2 ml cyclohexane. The two-phase culture was incubated at 37°C with shaking. Since the growth of cyclohexane-tolerant mutants was observed after 48 h, the aqueous medium phase was spread on LBGMg agar medium. Then, the agar surface was overlaid with a 3-mm-thick layer of cyclohexane. Strains that formed relatively large colonies on the agar after 48 h incubation at 25°C were randomly isolated as cyclohexane-tolerant mutants.

### PCR amplification and DNA sequencing of *acrR*, *marR*, and *soxR*

Chromosomal DNA of each bacterial isolate was used as the template for polymerase chain reaction (PCR) amplification. PCR was performed using PrimeSTAR® GXL DNA polymerase (Takara Bio, Kyoto, Japan). The primers used for PCR are listed in Table 2. The primer combinations used are as follows: acrR-F and acrR-R for the entire *acrR*; marR-F and marR-R for the entire *marR* and the operator region for *marR*; and soxR-F and soxR-R for the entire *soxR*. The PCR products were purified using the QiaQuick PCR purification kit (Qiagen, Hilden, Germany). Direct cycle sequencing in both directions was performed with the same sets of primers.

**Table 2:** Primers used in this study

**Primer**	**Sequence (5**^**′**^**to 3**^**′**^**)**	**Positions**^**a**^
acrR-F	AAACCCATTGCTGCGTTTAT	− 90 to − 71 bp of *acrR*
acrR-R	AAACCGCAAGAATATCACGA	+ 711 to + 730 bp of *acrR*
marR-F	CTGTTCATGTTGCCTGCCAG	− 321 to − 302 bp of *marR*
marR-R	CAGTCCAAAATGCTATGAATGG	+ 482 to + 503 bp of *marR*
soxR-F	TTTCTGATGGGACATAAATCTGCC	− 100 to − 77 bp of *soxR*
soxR-R	TGTGTTGACGTCGGGGGAAA	+ 537 to + 556 bp of *soxR*
acrA-F	CCAATTTGAAATCGGACACTCG	− 32 to − 11 bp of *acrA*
acrA-R	GCATGTCTTAACGGCTCCTG	+ 1200 to + 1219 bp of *acrA*
		
acrR-rpsL-neo-F	*GCTTCAGGATAATCCCGCTAACTTGAGGA CGAACTTCTGCGATCCGGTAG*GGCCTGGTGATGATGGCGGGATCG	− 376 to − 327 bp of *acrR* (indicated by italics), and − 138 to − 115 bp of *rpsL* in the *rpsL-neo* cassette (underlined)
acrR-rpsL-neo-R	*GCGATCGATTTTATCGAGGGTGGCTAATGTATCTGTCAGATCCTGCTG CA*TCAGAAGAACTCGTCAAGAAGGCG	+ 974 to + 1023 bp of *acrR* (indicated by italics), and + 750 to + 773 bp of *neo* in the *rpsL-neo* cassette (underlined)
marR-rpsL-neo-F	*AAACCGATAAACGCGACGATTAAGCCGCCTGCAATTCGCAGACCGGG AAT*GGCCTGGTGATGATGGCGGGATCG	− 477 to − 428 bp of *marR* (indicated by italics), and − 138 to − 115 bp of *rpsL* in the *rpsL-neo* cassette (underlined)
marR-rpsL-neo-R	*AAGAGAATAAGCGCAGCTGCTATTGCGGATGAAAGTGGTTTCATGATT GC*TCAGAAGAACTCGTCAAGAAGGCG	+ 863 to + 912 bp of *marR* (indicated by italics), and + 750 to + 773 bp of *neo* in the *rpsL-neo* cassette (underlined)
		
Repair-acrRIS-F	TGATCGTACTCTTGCTTACTGAT	− 575 to − 553 bp of *acrR*
Repair-acrRIS-R	ATCGTTTTGTGCGTTTTGCAAAT	+ 1202 to + 1224 bp of *acrR*
Repair-marR-F	TTTTCGCCTCCGGTGAATCA	− 527 to − 508 bp of *marR*
Repair-marR-R	AACTGGCTGCGTGGTTTGTT	+ 933 to + 952 bp of *marR*

^a^The positions of the primers are relative to the start codon of each gene.

### Site-directed point mutations in *E. coli* chromosome

For site-directed mutagenesis, the phage λ-based homologous recombination system (Red/ET counterselection Bac modification kit; GeneBridges, Heidelberg, Germany) was used to introduce an *rpsL-neo* cassette into *acrR* or *marR* of strain JA300 and to subsequently replace the cassette with an appropriate DNA fragment. Linear DNA fragments comprising the *rpsL-neo* cassette for the introduction of the *acrR* and/or *marR* region were obtained by PCR using the *rpsL*-neo template DNA (GeneBridges) as the template. The combinations of primers for the introduction of the *rpsL-neo* cassette were as follows: acrR-rpsL-neo-F and acrR-rpsL-neo-R for *acrR*, and marR-rpsL-neo-F and marR-rpsL-neo-R for *marR* (Table 2). These primers contain homology arms consisting of 50 nucleotides upstream and downstream from the targeted region and 24 nucleotides homologous to the *rpsL*-neo cassette. For all amplification, PrimeSTAR HS DNA polymerase (Takara Bio) with high fidelity was used. The *acrR* or *marR* region into which the *rpsL-neo* cassette was introduced was replaced with corresponding DNA fragments containing a mutated gene of *acrR* or *marR* from strain CH7. The DNA fragments for the replacement of the *rpsL-neo* cassette were obtained by PCR using chromosomal DNA prepared from strain CH7 as the template. The combinations of primers for replacement of the *rpsL-neo* cassette are as follows: Repair-acrRIS-F and Repair-acrRIS-R for *acrR*, and Repair-marR-F and Repair-marR-R for *marR*.

Transformations of cells by the introduction of linear DNA fragments for recombination were performed by electroporation according to the protocol recommended by the technical manual of the Bac modification kit (Gene Bridges). Briefly, the *E. coli* strain carrying pRed/ET (Gene Bridges) was cultivated in 1.4 ml of LB medium at 30°C. At an absorbance of 0.3 (600 nm), freshly prepared L-arabinose was added (0.35% wt/vol, final concentration) to the culture inducing *red*γβα*/recA* expression, and expression was continued at 37°C. After 1 h, the cells were harvested by centrifugation, washed with 10% (vol/vol) ice-cold glycerol, and resuspended in a final volume of 30 μl in 10% (vol/vol) ice-cold glycerol. DNA fragments (100 to 200 ng) were then added to the sample. Subsequently, the sample was transferred to electroporation cuvettes, and electroporation was carried out with an Electroporator 2510 (Eppendorf, Hamburg, Germany) at 1350 V, 10 μF, and 600 Ohms. The cells were immediately removed from the cuvettes by mixing with 1 ml LB medium, and then were incubated at 37°C for 2 h. The cells were plated on LB agar containing the appropriate antibiotics.

Recombination events were confirmed by PCR and DNA sequencing of the *acrR* and *marR* genes*.* The combinations of primers used are as follows: acrR-F and acrR-R for *acrR*, and marR-F and marR-R for *marR.*

### Disruption of *acrA* in strain JA300 and JA300 *acrR*IS *marR*

JA300Δ*acrA* and JA300Δ*acrA acrR*IS *marR* were constructed by P1 transduction of kanamycin resistance, with strain JW0452 as the donor. Disruption of the gene was confirmed by PCR analysis using chromosomal DNA prepared from JA300Δ*acrA* or JA300Δ*acrA acrR*IS *marR* as the template. The combination of primers used was acrA-F and acrA-R (Table 2). Consistent with the expected values, the size of the amplified product (*acrA*) from JA300 was 1,252 bp, and those from kanamycin-resistance cassette transductants were approximately 1.5 kb.

### Measurement of the organic solvent tolerance of *E. coli*

Cultures of *E. coli* strains in LBGMg medium (optical density at 660 nm [OD_660_], 0.4 to 0.6) were diluted with 0.8% saline by serial 10-fold dilutions. Each suspension was plated on LBGMg agar. The agar surface was overlaid with a 3-mm-thick layer of an organic solvent. The approximate frequency at which the cells formed colonies on the agar was estimated after 48 h incubation at 25°C.

### Quantitation of organic solvent accumulation in *E. coli* cells

The organic solvent accumulation in *E. coli* cells was quantitated as described previously (Doukyu et al. 2012 ). An organic solvent was added to a suspension of *E. coli* cells harvested during the late exponential phase of growth (OD_660_, 1.5 to 2.0). The suspension was centrifuged after incubation with the solvent for 30 min. After separation from the medium layer, the solvent layer was removed by aspiration. The medium was disposed of by decanting. The cell pellet was recovered and suspended in 1.0 ml of 0.9% NaCl–10 mM MgSO_4_. A 0.5 ml portion of the suspension was then transferred to an Eppendorf tube, and the remaining portion was kept to measure the protein content. The 0.5 ml cell suspension was extracted with 2.0 ml of CHCl_3_ by vigorous shaking for 90 min at 25°C in a shaker (Handless shaker SHK-COCK; Asahi Technoglass, Tokyo, Japan). The amount of organic solvent in the CHCl_3_ extract was measured using a gas chromatography–mass spectrometry apparatus (GCMS-QP2010 Plus; Shimadzu, Kyoto, Japan) with an Rtx®-624 column (30 m by 0.25 mm inside diameter, 1.4 μm film thickness; Restek, PA, USA). The column was eluted with helium gas at a flow rate of 1.69 ml/min. The inlet was set at 150°C, and the oven was programmed as follows: 50°C for 5 min, then increasing by 5°C per minute to 100°C. The total ion chromatogram of an organic solvent was detected with a mass selective detector.

### Protein content

Protein content was measured by the method of Lowry et al. (Lowry et al. 1951 ).

### Antibodies against AcrA, AcrB, and TolC

Antibodies against AcrA were obtained as described previously (Doukyu et al. 2012 ). Polyclonal antibodies against AcrB and TolC were raised against synthetic peptides corresponding to regions of AcrB and TolC. These antibodies contained an N-terminal cysteine (C + in the peptides shown below) for the conjugation of keyhole limpet hemocyanin. The peptide sequences were as follows: a synthetic peptide for anti-AcrB antibodies, C + KNEDIEHSHTVDHH (corresponding to residues 1036 to 1049) and a peptide for anti-TolC antibodies, C + ARTTTSNGHNPFRN (corresponding to residues 480 to 493). The conjugated peptides were injected into rabbits, and polyclonal antibodies were purified from serum using a Melon gel immunoglobulin G spin purification kit (Thermo Fisher Scientific, Rockford, IL, USA). These antibodies were used to detect AcrA, AcrB, and TolC in the immunoblotting analyses of this study.

### Immunoblotting analyses

*E. coli* was grown in LBGMg medium. The cells were harvested during the exponential phase of growth (OD_660_, 0.6) by centrifugation (5,000 × *g* for 10 min at 4°C). The cells were suspended in cold 10 mM Tris–HCl buffer (pH 8.0) and broken by sonication in an icewater bath. Ten micrograms of total cell lysate protein in the supernatant was separated by sodium dodecyl sulfate-polyacrylamide gel electrophoresis (SDS-PAGE) in a 12.5% separating gel ( Laemmli 1970 ). The gels were then transferred to a polyvinylidene difluoride membrane (Immobilon-P; Millipore, Bedford, MA, USA), electrophoretically by the application of 50 V for 30 min in Tris-glycine-methanol buffer (25 mM Tris, 192 mM glycine, 20% [vol/vol] methanol [pH 8.3]). The membrane was blocked overnight at room temperature in Tris-buffered saline (TBS; 0.15% NaCl, 10 mM Tris–HCl, pH 7.4) containing 3% gelatin, washed three times in wash buffer (0.05% Tween 20 in TBS), and hybridized at room temperature with the relevant antibody. The membrane was then probed for 1 h with goat anti-rabbit horseradish peroxidase (Bio-Rad, Hercules, CA, USA). The bands were visualized by use of an alkaline phosphatase color development kit (Bio-Rad). The AcrA, AcrB, and TolC expression levels were quantified by gel analysis software UN-SCAN-IT (Silk Scientific, Orem, UT, USA).

## Results

### Isolation of cyclohexane-tolerant mutants of *E. coli* JA300

*E. coli* K-12 strain JA300 is tolerant to an organic solvent with a log*P*_ow_ value of more than 4 and is highly sensitive to cyclohexane (log*P*_ow_, 3.4) ( Aono 1998 ). Cyclohexane-tolerant mutants that formed colonies on the LBGMg agar medium overlaid with cyclohexane were isolated from strain JA300. Spontaneous cyclohexane-tolerant mutants from strain JA300 appeared on the LBGMg agar medium overlaid with cyclohexane at a frequency of 1.6 × 10^-6^. In the present study, to efficiently isolate mutants with high-level organic solvent tolerance, the parent strain JA300 was precultured in the LBGMg liquid medium overlaid with cyclohexane. The viable cell density in the preculture increased to 2.2 × 10^5^ /ml after 48 h incubation. The preculture was spread on the agar medium, and then the agar surface was overlaid with cyclohexane. After 48 h, mutants appeared on the agar medium with cyclohexane at a frequency of 5.0 × 10^-3^. The organic solvent tolerances of about 100 isolates randomly selected were investigated by measuring colony-forming efficiencies on an agar plate overlaid with cyclohexane. As a result, eight mutants (CH1 to CH8) exhibiting relatively high colony-forming efficiencies on the agar medium with cyclohexane were selected and used in further experiments.

### Organic solvent tolerances of the isolated mutants

**Figure 1:**
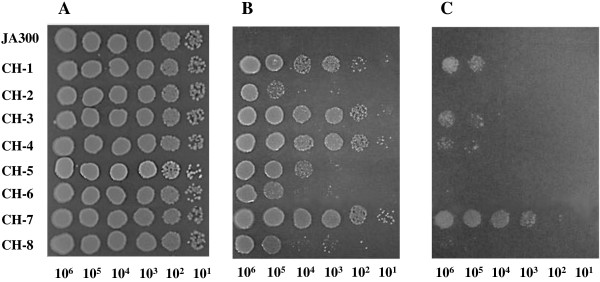
**Colony-forming efficiency of organic solvent-tolerant mutants from *E. coli *JA300 on LBGMg agar medium in the absence of an organic solvent (A) and in the presence of cyclohexane (B) or cyclohexane and *p*-xylene (7:3 vol/vol mixture) (C).** Each strain was spotted at a tenfold dilution and incubated at 25°C for 48 h.

The organic solvent tolerances of the mutants (CH1 to CH8) were investigated by measuring the colony-forming efficiency of each mutant on an agar plate overlaid with pure cyclohexane and a mixture of cyclohexane and *p*-xylene (7:3 vol/vol mixture) (Figure 1). *p*-Xylene (log*P*_ow_, 3.1) shows higher toxicity to cells than cyclohexane. All strains formed colonies in all spots on the plate without any solvent. The parent strain JA300 did not form any colony in the presence of cyclohexane and the solvent mixture. All mutants formed colonies in the presence of cyclohexane, although the colony-forming efficiencies differed among the mutants. Strains CH1, CH3, CH4, and CH7 exhibited relatively high organic solvent tolerance in the presence of cyclohexane. Strains CH2, CH5, CH6, and CH8 did not form any colonies in the presence of the solvent mixture. On the other hand, strain CH7 formed colonies even in spots containing 10^3^ cells in the presence of the solvent mixture and therefore showed the highest colony-forming efficiency among all isolates.

### Identification of mutations in *acrR*, *marR*, and *soxR*

The nucleotide sequences of entire *acrR*, *marR*, and *soxR* genes in organic solvent-tolerant mutants were determined by DNA sequencing to identify mutations in these genes. The DNA sequences were analyzed by comparison with those of the JA300 parent strain. These mutations are summarized in Table 3. The sequences of *acrR*, *marR*, and *soxR* genes in strain JA300 were identical to those present in the *E. coli* K-12 strain MG1655 genome deposited in GenBank (accession number NC_000913.2). None of the mutants carried a mutation in *soxR*. Seven of the eight cyclohexane-tolerant mutants carried four different mutations in *marR*. Among these seven *marR* mutants, three mutants (CH1, CH3, and CH4) had point mutations causing an amino acid substitution. These mutations were G116C (CH1), L78M (CH3), and R94L (CH4). Four strains (CH5, CH6, CH7, and CH8) had the same mutation, which would cause premature termination of the *marR* translation. This was caused by a nonsense codon (E109 → TAA stop). This mutation was named *marR109*.

**Table 3:** **Mutations in*marR*and*acrR*of cyclohexane-tolerant*E. coli*mutants**

**Strain**					
	DNA position^a^ (Codon substitution)	Amino acid substitution		DNA position^a^ (Codon substitution)	Amino acid substitution or insertion
CH1	346 (GGC → TGC)	G116C		122 (GCT → GAT)	A41D
CH2		None		3 (ATG → ATA)	M1I
CH3	232 (CTG → ATG)	L78M			None
CH4	281 (CGC → CTC)	R94L			None
CH5	325 (GAA → TAA)	E109 → TAA stop			None
CH6	325 (GAA → TAA)	E109 → TAA stop			None
CH7	325 (GAA → TAA)	E109 → TAA stop		220	Insertion of IS*5*
CH8	325 (GAA → TAA)	E109 → TAA stop			None

^a^DNA positions of the mutations are relative to the start codon of each gene.

**Figure 2:**
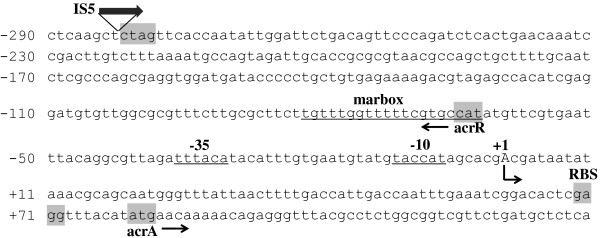
**Nucleotide sequence of the *acrAB *regulatory region.** The transcription initiation site (+ 1) of the *acrAB* operon is capitalized and indicated by a bent arrow. The CTAG IS*5* target site, the putative ribosome binding site (RBG) for *acrA*, and the ATG start codons for *acrR* and *acrAB* are shaded. The promoter regions (− 10 and − 35) (Martin et al. 1999 ) and marbox (Su et al. 2007 ) are underlined. The bold arrow above the sequence indicates the insertion site of IS*5*.

Three mutants (CH1, CH2, and CH7) carried mutations in *acrR*. Strain CH2 had a mutation only in *acrR*. This mutation was found in the translation initiation codon (M1I). The other two mutants (CH1 and CH7) each carried mutations in both *marR* and *acrR*. Strain CH1 had a point mutation altering one amino acid residue (A41D) in *acrR*. Strain CH7 contained an 1195-bp insertion sequence (IS*5*) ( Sawers 2005 ) integrated within *acrR* (Figure 2). This sequence was inserted at 220 bp downstream from the initiation codon of the *acrR* gene, accompanied by the doubling of the tetramer CTAG. The *ins5A* promoter was oriented in the same direction as the *acrAB* operon.

### Organic solvent tolerances of mutants carrying mutations in *marR* and/or *acrR* derived from strain CH7

**Figure 3:**
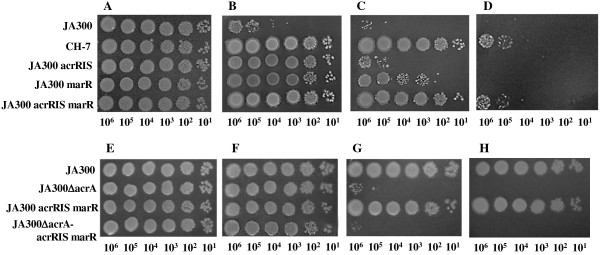
**Colony-forming efficiency of *E. coli *JA300 mutants on LBGMg agar medium with or without an organic solvent.** Colony-forming efficiency of *acrR* and/or *marR* mutants on the agar medium in the absence of an organic solvent (**A**) and in the presence of *n*-hexane (**B**), cyclohexane (**C**), or cyclohexane and *p*-xylene (6:4 vol/vol mixture) (**D**). Colony-forming efficiency of *acrA* disruptants of JA300 and JA300 *acrR*IS *marR* on LBGMg agar medium in the absence of an organic solvent (**E**) and in the presence of decane (**F**), nonane (**G**), or octane (**H**). Each strain was spotted at a tenfold dilution and incubated at 25°C for 48 h.

Strain CH7 had a nonsense mutation in *marR* and an insertion of IS5 in *acrR*. To clarify the involvement of these mutations in organic solvent tolerance, the *marR* and/or *acrR* regions were introduced into the *E. coli* JA300 chromosome by site-directed mutagenesis using λ red-mediated homologous recombination. Consequently, JA300 mutants carrying *acrR*::IS*5*, *marR109*, or both mutations were constructed and named JA300 *acrR*IS, JA300 *marR*, or JA300 *acrR*IS *marR*, respectively. The colony-forming efficiencies of these constructed mutants in the presence of organic solvents were compared with those of the parent strain JA300 and strain CH7 (Figure 3). All strains formed colonies in all spots on the plate without any solvent. The parent strain JA300 formed colonies in the spots containing 10^5^-10^6^ cells in the presence of *n*-hexane (log*P*_ow_, 3.9). However, strain JA300 hardly formed colonies on the plate overlaid with pure cyclohexane and did not form any colony on the plate with cyclohexane and *p*-xylene (6:4 mixture). In contrast, the colony-forming efficiencies of the constructed mutants in the presence of the organic solvents were increased in the following order: JA300 *acrR*IS < JA300 *marR* < JA300 *acrR*IS *marR*. JA300 *acrR*IS *marR* exhibited about 10^2^- and 10^4^-fold higher colony-forming efficiencies than those of JA300 *acrR*IS and JA300 *marR*, respectively, in the presence of cyclohexane. JA300 *acrR*IS and JA300 *marR* did not form any colony on the plate overlaid with the solvent mixture, although JA300 *acrR*IS *marR* formed colonies in spots containing 10^5^-10^6^ cells in the presence of the solvent mixture. JA300 *acrR*IS *marR* showed similar colony-forming efficiencies as strain CH7 in the presence of the solvents tested.

**Figure 4:**
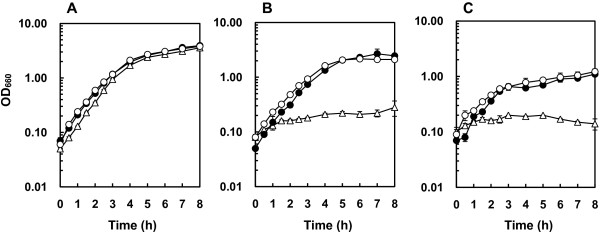
**Growth of *E*. *coli *JA300, JA300 *acrR*IS *marR*, and CH7 in LBGMg liquid medium in the absence of an organic solvent (A) and in the presence of *n*-hexane (B) and cyclohexane (C).** A 100-μl culture of overnight-grown *E. coli* strain was inoculated to 10 ml of fresh LBGMg liquid medium overlaid with an organic solvent. This two-phase culture was incubated at 30°C. Growth was monitored by measuring turbidity (OD_660_). Values indicate means of results and standard deviations of results from three independent experiments. Symbols: (△) JA300; (·) JA300 *acrR*IS *marR*; (○) CH7.

The cell growth of JA300, JA300 *acrR*IS *marR*, and CH7 in the LBGMg liquid medium in the presence of *n*-hexane or cyclohexane was also examined by measuring turbidity (Figure 4). No significant difference was found between the growth of these strains in the absence of organic solvents. The growth of JA300 was highly suppressed by the addition of organic solvents. In contrast, JA300 *acrR*IS *marR* and CH7 were able to grow in the presence of these solvents, although the growth rates and yields of these strains were partially reduced by the addition of these solvents as compared to that without any solvent.

### AcrA, AcrB, and TolC levels in organic solvent-tolerant mutants

**Figure 5:**
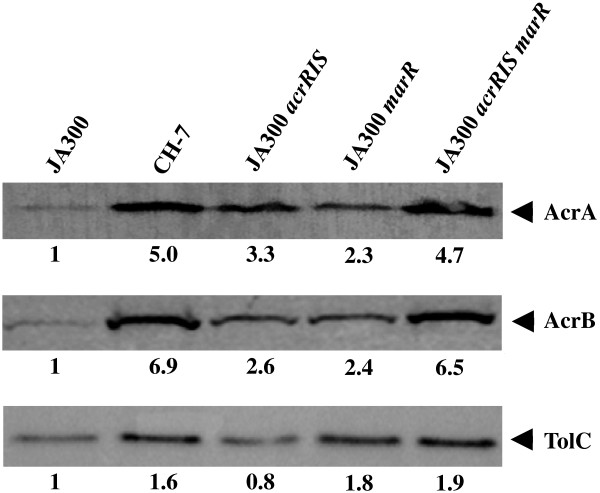
**Western blot analysis of AcrA, AcrB, and TolC expression.** Total cell lysate proteins of JA300 (lane 1), CH7 (lane 2), JA300 *acrR*IS (lane 3), JA300 *marR* (lane 4), and JA300 *acrR*IS *marR* (lane 5) were separated by sodium dodecyl sulfate-polyacrylamide gel electrophoresis and probed with polyclonal anti-AcrA, AcrB, and TolC antibodies, respectively. The expression ratio compared to the AcrA level of JA300 is shown below each lane.

Mutations in *marR* can increase the expression levels of AcrAB and TolC proteins, which are components of the AcrAB-TolC efflux pump ( Barbosa and Levy 2000 ). In addition, mutations in *acrR* can enhance the expression of AcrAB (Ma et al. 1996 ; Webber et al. 2005 ). Levels of AcrA, AcrB, and TolC in JA300, CH7, JA300 *acrR*IS, JA300 *marR*, and JA300 *acrRIS marR* were investigated by immunoblotting analysis (Figure 5). Both the AcrA and AcrB levels in JA300 *acrR*IS were about threefold higher than those in JA300. However, the TolC level in JA300 *acrR*IS was similar to that in JA300. The levels of AcrA, AcrB, and TolC in JA300 *marR* were about twice those in JA300. JA300 *acrR*IS *marR* exhibited higher expression levels of AcrA and AcrB compared to those of JA300 *acrR*IS and JA300 *marR*, but the TolC level in JA300 *acrR*IS *marR* was similar to that in JA300 *marR*. The levels of these three proteins in JA300 *acrR*IS *marR* were similar to those in strain CH7.

### Organic solvent tolerance of *acrA*-disruptant

Since the *marR109* mutation can influence the expression levels of many *mar* regulon genes, including *acrAB* and *tolC* ( Barbosa and Levy 2000 ), it was possible that *mar* regulon genes other than *acrAB* and *tolC* could be involved in the improved organic solvent tolerance in JA300 *acrR*IS *marR*. To clarify the extent to which the AcrAB-TolC pump contributed to organic solvent tolerance in JA300 *acrR*IS *marR*, a JA300 *acrR*IS *marR*-based *acrA*-disruptant, JA300Δ*acrA acrR*IS *marR*, was constructed and then the organic solvent tolerance of this *acrA*-disruptant was compared to that of JA300Δ*acrA* (Figure 3). The organic solvent tolerance of JA300Δ*acrA* was similar to that of the previously reported JA300-based *acrAB* disruptant ( Tsukagoshi and Aono 2000 ). JA300 was tolerant to nonane (log*P*_ow_, 5.5) and octane (log*P*_ow_, 4.9). In contrast, JA300Δ*acrA acrR*IS *marR* became sensitive to these solvents. The tolerance level of JA300Δ*acrA acrR*IS *marR* was similar to that of JA300Δ*acrA*.

### Accumulation of an organic solvent in *E. coli* incubated in a two-phase culture system

It has been reported that organic solvent-tolerant *E. coli* strains in a two-phase culture system maintained low intracellular levels of organic solvents ( Tsukagoshi and Aono 2000 ; Shimizu et al. 2005 ; Doukyu et al. 2012 ). The amounts of *n*-heptane (log*P*_ow_, 4.2), *n*-hexane (log*P*_ow_, 3.9), or cyclohexane (log*P*_ow_, 3.4) accumulated in *E. coli* cells were investigated (Table 4). *E. coli* cells of JA300, CH7, JA300 *acrR*IS, JA300 *marR*, or JA300 *acrR*IS *marR* were incubated for 30 min in the presence of organic solvents. The intracellular solvent levels of JA300 *acrR*IS and JA300 *marR* were similar to or slightly lower than those of the JA300 parent strain. On the other hand, the intracellular solvent levels of CH7 and JA300 *acrR*IS *marR* were remarkably lower than those of the JA300 parent strain. The amounts of *n*-heptane, *n*-hexane, and cyclohexane in CH7 were 6%, 20%, and 30% of those for JA300, respectively. On the other hand, the amounts of *n*-heptane, *n*-hexane, and cyclohexane in JA300*acrR*IS *marR* were 2%, 19%, and 22% of those for JA300, respectively. Thus, the intracellular amounts of organic solvents in JA300 *acrR*IS *marR* exhibited levels similar to those in CH7.

**Table 4:** **Accumulation of organic solvents in *E. coli *cells in a two-phase system**

**Strain**			
	*n*-Heptane	*n*-Hexane	Cyclohexane
JA300	0.060 ± 0.002	0.54 ± 0.01	1.3 ± 0.1
JA300 *acrR*IS	0.051 ± 0.037	ND^b^	1.2 ± 0.1
JA300 *marR*	0.049 ± 0.010	ND^b^	1.0 ± 0.1
JA300 *acrR*IS *marR*	0.0010 ± 0.0006	0.10 ± 0.01	0.29 ± 0.01
CH7	0.0033 ± 0.0002	0.11 ± 0.01	0.39 ± 0.01

^a^*E. coli* strains grown in LBGMg medium were exposed to organic solvents in the two-phase system and incubated for 30 min as described in Materials and Methods. Values indicate means of results and standard deviations of results from three independent experiments.

^b^ND, not determined.

## Discussion

In the present study, we isolated cyclohexane-tolerant mutants from cyclohexane-sensitive *E. coli* K-12 strain JA300 and investigated whether or not these mutants carried mutations in regulatory genes *marR*, *soxR*, and *acrR*. Most of the mutants carried mutations in *marR*. Three of the seven mutations found in *marR* caused amino acid substitutions in MarR at the amino acid positions of L78, R94, and G116, and four of the seven mutations led to a translation termination codon at the position of E109 (*marR109* mutation). These MarR mutations lie within the region spanning amino acids 61 to 121 in MarR, which are required for DNA binding activity (Alekshun et al. 2001 ). The L78 and R94 residues are highly conserved amino acids in many homologs, although the amino acid at the position of G116 is not conserved (Alekshun et al. 2001 ). The *marR109* mutation led to the truncation of 35 C-terminal amino acids in MarR. A previous study showed that the C-terminal region contributes to dimer formation (Notka et al. 2002 ). In any case, the MarR mutations found in the present study are considered to lead to the loss of repressive function. Three mutants isolated in this study carried different mutations in *acrR*. Two mutations in *acrR* caused amino acid substitutions in AcrR at the amino acid positions of M1 and A41. The mutation in the translation initiation codon (M1I) will cause the complete inhibition of AcrR translation. The A41 residue positioned within the helical region (α3) forms part of a typical helix-turn-helix motif involved in DNA binding (Li et al. 2007 ). Thus, the A41D mutation will abolish AcrR’s repressive activity. Many IS elements have been shown to activate or inactivate the expression of neighboring genes. In strain CH7, the IS*5* inserted within *acrR* seemed to activate the expression of *acrAB* through the disruption of transcriptional repression by AcrR. The alteration of organic solvent tolerances of several *E. coli* mutants by IS integration has been reported. *E. coli* OST4251 became sensitive to *n*-hexane because IS*2* and IS*5* became integrated upstream from the *imp*/*ostA* gene, which is involved in organic solvent sensitivity (Abe et al. 2003 ; Ohtsu et al. 2004 ). The hypersensitivity of an *E. coli acrB* disruptant to organic solvents was suppressed by the integrational activation of the *acrEF* operon with an IS*1* or IS*2* element (Kobayashi et al. 2003 ).

In this study, two mutants (CH1 and CH7) carried mutations in both *marR* and *acrR*. These mutants exhibited higher organic solvent tolerances than other isolates (Figure 1). In particular, strain CH7 containing *marR109* and *acrR*::IS showed the highest organic solvent tolerance among all isolates. In fluoroquinolone-resistant clinical and veterinary isolates of *E. coli*, a number of mutations were found in *soxR* as well as *marR* and *acrR* (Wang et al. 2001 ; Webber and Piddock 2001 ; Komp Lindgren et al. 2003 ). In the present study, however, there was no mutation in *soxR* in cyclohexane-tolerant mutants.

It was possible that unidentified mutations other than *marR109* and *acrR*::IS*5* might influence organic solvent tolerance in strain CH7. To clarify the effect of the *marR109* and/or *acrR*::IS*5* mutations on organic solvent tolerance in *E. coli*, we constructed JA300 *acrR*IS, JA300 *marR*, and JA300 *acrR*IS *marR*. A comparison of the tolerances in these mutants and in strain CH7 revealed that the improved organic solvent tolerance in strain CH7 was caused by a synergistic effect of the double mutations of *marR* and *acrR.*

The AcrAB-TolC efflux pump is involved in organic solvent tolerance in *E. coli* ( Tsukagoshi and Aono 2000 ). The order of organic solvent tolerances of JA300 *acrR*IS, JA300 *marR*, and JA300 *acrR*IS *marR* was comparable to the order of the expression levels of AcrAB and TolC (Figures 3 and 5). The expression levels of AcrA and AcrB proteins in JA300 *acrR*IS were similar to, or slightly higher than, the levels in JA300 *marR*. However, the extent of improvement in organic solvent tolerance in JA300 *acrR*IS was lower than that in JA300 *marR* because the disruption of *acrR* did not influence the expression level of TolC. JA300 *acrR*IS *marR* and CH7 equally enhanced the expression levels of AcrAB and TolC compared to JA300 *acrR*IS and JA300 *marR*. In addition, the intracellular solvent levels of JA300 *acrR*IS *marR* and CH7 were similarly kept lower than those of JA300 *acrR*IS, JA300 *marR*, and JA300. These results suggested that the improved organic solvent tolerance in JA300 *acrR*IS *marR* and CH7 was a result of enhanced solvent-efflux activity by the overexpressed AcrAB-TolC pump. To clarify the contribution of the AcrAB-TolC pump to organic solvent tolerance in JA300 *acrR*IS *marR*, an *acrA*-disruptant (JA300Δ*acrA acrR*IS *marR*) was constructed and its organic solvent tolerance was compared to that of JA300Δ*acrA* (Figure 3). JA300Δ*acrA acrR*IS *marR* became as sensitive to organic solvents as JA300Δ*acrA*. This result indicated that the AcrAB-TolC pump is essential for JA300 *acrR*IS *marR* to acquire high-level organic solvent tolerance. In addition, it suggested that the *mar* regulon genes other than *acrAB* and *tolC* are barely involved in organic solvent tolerance in JA300 *acrR*IS *marR*.

Organic solvent-tolerance levels of various mutants and recombinants from strain JA300 have been investigated by measuring the colony-forming efficiencies of mutants on an LBGMg agar plate overlaid with organic solvents. Overexpression of the *marA* gene has been shown to raise the organic solvent tolerance of *E. coli* (Asako et al. 1997 ; Shimizu et al. 2005 ). JA300 overexpressing the *marA* gene formed colonies in spots containing more than 10^6^ cells in the presence of cyclohexane (Shimizu et al. 2005 ). We previously reported that the organic solvent tolerance of strain JA300 significantly improved the double disruptions of *marR* and *proV* (Doukyu et al. 2012 ). JA300Δ*proV*Δ*marR* formed colonies in spots containing more than 10^5^ cells in the presence of cyclohexane and thus exhibited higher organic solvent-tolerance levels than JA300 overexpressing the *marA* gene. In the present study, JA300 *acrR*IS *marR* showed 10^4^-fold higher colony-forming efficiencies in the presence of cyclohexane than JA300Δ*proV*Δ*marR*.

In the present study, we clarified that only two mutations in regulatory genes in *acrR* and *marR* confer high-level organic solvent tolerance on *E. coli*. Owing to the wealth of genetic and metabolic knowledge associated with *E*. *coli*, organic solvent-tolerant *E. coli* can be a convenient and efficient catalyst when it is used as a host expressing enzymes that are useful for producing valuable chemicals in two-phase systems employing organic solvents. The present findings are expected to provide valuable knowledge for increasing organic solvent-tolerance levels in *E. coli* to improve the usability of whole-cell biocatalysts in two-phase systems.

## Competing interests

The authors declare that they have no competing interests.
